# Making sense of meta-analysis in medical education research

**DOI:** 10.5116/ijme.5c4d.0078

**Published:** 2019-01-30

**Authors:** Mohsen Tavakol

**Affiliations:** 1 School of Medicine, Medical Education Centre, The University of Nottingham, UK

**Keywords:** Meta-analysis, medical education research, effect size

## Introduction

Interest in and use of experimental and quasi-experimental studies has increased among medical educators. These studies may be focused on a cause and effect relationship if they are rigorously conducted. Put another way; the medical education researcher manipulates or controls the independent variable (cause) in order to evaluate its impact on the dependent variable or outcome (effect). It is difficult to establish a cause and effect relationship in medical education research because the researcher is unable to control all covariables (confounding/intervening variables) that can influence the outcome of the study.  Campbell and Stanley described confounding variables as threats to internal and external validity.^1^ Internal validity refers to the degree to which changes in the outcome(s) (the dependent variable(s)) of the study can be accounted for by the independent variable(s).  Factors that may be considered as threats to internal validity are not part of the independent variables in an experimental study, but they can have a significant effect on the dependent variable (s) (outcome). Indeed, these factors may account for the results of the study, not the independent variable (s) (intervention (s)) of interest. External validity is focused on the extent to which the results of the study can be generalised to the target population. Threats to internal and external validity can undermine the quality of a meta-analysis which is grounded in a systematic review of the relevant literature.   The methodological quality of experimental studies in medical education research may be in error. Given the possibility of methodological errors in experimental or quasi-experimental studies, authors of meta-analyses should first critically appraise the quality of all relevant studies comprised in the meta-analysis. For further discussion of criteria for experimental and quasi-experimental studies, I refer the interested readers to more extended discussions of quantitative and qualitative methods in medical education research.^2^^,^^3^

### Intervention effect

One of the criteria for conducting an experimental study is to manipulate the experimental independent variable. By manipulating the independent variable, we mean that the researcher controls the independent or experimental variable to evaluate its impact on the dependent variable (s) (outcome(s)). In medical education research, the experimental variable is typically an education intervention, for instance, the impact of simulation-based education on the development of clinical reasoning or the performance of medical students. The researcher conducting an experimental study manipulates the intervention of interest (e.g., simulation-based training) by administrating it to some students (the experimental group) and not to other students (control group). The control group usually receives a routine intervention, e.g., the usual teaching. After randomly assigning students into two groups, the experimental and the control groups, both groups take a pre-test as a basis for comparison of their performance on the pre-test with a post-test, which is given after the experimental group receives the intervention of interest and the control group receives a routine treatment. Using a measurement instrument, to assess the performance of both groups before and after the intervention and the routine treatment, is a pre-test-post-test control group design. If we assume that the collected data for measuring the performance of students is a continuous measure, we calculate the means and standard deviation of student performance. Using inferential statistics, the researcher is able to determine the impact of the intervention of interest on the performance of students. The fundamental data analysis is to calculate effect size indices, which inform us about the magnitude of the effect of an intervention (e.g., simulation) on particular outcomes (e.g., student performance). The effect size indicates the magnitude of differences in two means, e.g., the difference between interventional and control group means on student performance. Effect sizes indicate whether the differences are important. Effect sizes are essential for conducting meta-analyses. Sometimes the outcomes of studies with experimental designs are dichotomous and meta-analysts use the Odds Ratios (OR) or Risk Ratios (RR). In non-experimental studies, they may use the Pearson correlation coefficient (i.e., Pearson’s r) to show the strength and direction of an effect.

The purpose of this introductory guide is to show how a meta-analysis works in the context of medical education research using experimental studies. The purpose of this article is to introduce standards and methods for meta-analysis for experimental studies in order to synthesise data from the primary research studies using meta-analysis and related statistics.  This paper does not deal with the primary steps of a meta-analysis, i.e., how to address a problem, how to design a meta-analysis, how to appraise the quality of primary research studies, and how to extract and code data for analysis. Once these steps are completed, the meta-analysis is performed. Interested readers may refer to systematic review texts to conduct a thorough meta-analysis.

### What is meta-analysis?

Meta-analysis is a statistical analysis that provides for a scholar to combine and synthesise the results of multiple primary research studies in order to minimise uncertainty and disagreement.^4^^-^^6^ The rationale associated with meta-analysis is to increase overall sample sizes, which in turn could enhance the statistical power analysis as well as the precision of intervention effects.^7^

Meta-analysis consists of two steps. In the first step, the scholar calculates the effect size and variance, with 95% Confidence Intervals (CIs) for each study. The narrower the CI, the greater will be the precision of the study. The larger the study, the more precise will be the effect size. Next, in the second step, the scholar calculates a summary of effect size (a pooled effect estimate) which is considered to be a weighted mean of the individual effects. Therefore, the term weight plays a vital role in meta-analysis based on multiple studies.  We can calculate the weight of each study by the inverse of variance (the square of the standard error) of the intervention effect.  High study weights will significantly contribute to the weighted mean. As standard errors are low in larger studies, they yield higher weights in comparison with the weights in smaller studies.  A critical point in meta-analysis is that the effects are not heterogeneous across a collection of the primary research studies, which is a matter of the heterogeneity, discussed below.

### Heterogeneity in effect sizes

Heterogeneity is concerned with the variability or scattering of effect sizes between and among studies. Considering if the overall mean effect size for the effectiveness of simulation training on student performance is medium, but you observe that the effect sizes are significantly different across a set of studies, i.e., some have small effect sizes, and some have a medium or large effect sizes. Scholars using meta-analysts assess and report the heterogeneity of results, using statistical techniques. The heterogeneity of results helps us to decide whether to continue with the meta-analysis of a set of primary research studies.  Besides, the heterogeneity of results directs us towards the use of statistical models that should be used for the analysis, which is discussed below. We can detect the heterogeneity of the results using a visual assessment of forest plots. Also, forest plots contain other useful information, which is discussed below.

### Forest plot

**Figure 1:**
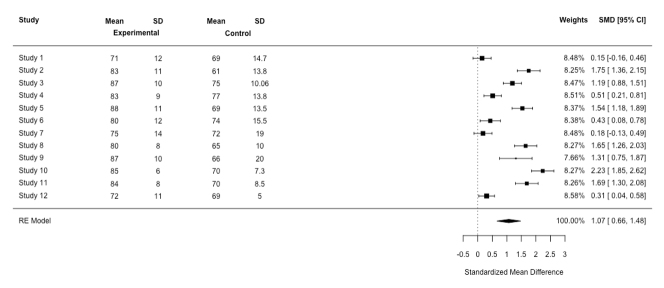
Forest plot for the simulation-based training meta-analysis based on a random-effect (RE) model

The results of a meta-analysis are usually illustrated using a forest plot. To describe the forest plot, suppose that we have systematically reviewed 1^2^ articles to investigate the effect of simulation-based training on student performance. Suppose further that authors of each study have used a different scale to measure student performance (a continuous outcome). The first step is to compute the mean, standard deviations and effect sizes for the experimental group and the control group for each study. Figure 1 shows a forest plot for this hypothetical study. It should be emphasised that when we are interested in measuring the pre and post-test performance of two independent groups, e.g., experimental group and control, the best statistics for calculating the effect size is the standardise mean difference (SMD).^5^ This is due to the fact that the researchers might have used a different scale to measure the student performance.

### Description of the forest plot

As we can see from Figure 1, the first column shows the studies of interest. The last column shows the effect sizes, i.e., SMD, and confidence intervals. Each study has its own line, i.e., its raw data, SMD and confidence interval. This information is also graphically presented. For example, the largest SMD can be found in study 10 (2.23) and the smallest one can be found in study 1 (0.15). The black box corresponds to the point estimate (the value of SMD) of the individual studies, e.g., in study 12 it is 0.31. The size of the box represents the weight given to the study in the meta-analysis.  The horizontal lines (whiskers) of boxes indicate the 95% confidence interval. The larger the box, the larger the weight. The vertical line is called the ‘no effect' line, i.e., SMD is equal to zero. When the horizontal lines (whiskers) of boxes cross the vertical line, this indicates there is no statistically significant difference between the experimental group and the control group, e.g., studies 1 and 7. Stated in another way, if the confidence interval encompasses 0, then there is no statistical difference between the performance of the experimental group and the control group. At the bottom, the diamond reflects the summery effect in meta-analysis. The center of the diamond indicates the combined experimental effect, i.e., 1.07, and its ends indicates the 95% confidence interval, i.e., [0.66, 1.48]. If the diamond is located at the right of the vertical line (the no effect line), then one can conclude that student performance will increase in the experimental group (those who have received simulation training) as compared with the control group.

**Figure 2:**
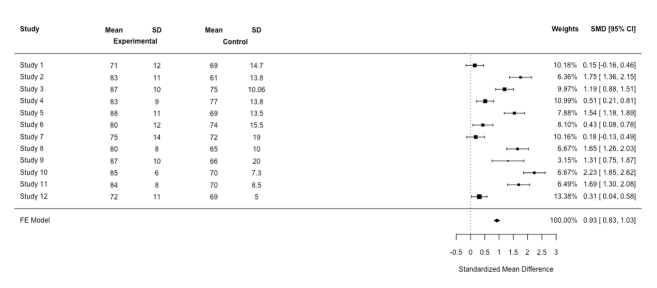
Forest plot for the simulation-based training meta-analysis based on a fixed-effect (FE) model

If the diamond crosses the vertical line, one can conclude that there is no statistically significant difference in performance between the control group and the experimental group. In Figure 1, the diamond does not cross the vertical line. In other words, the confidence interval for the SMD does not encompass 0, meaning that the difference between the experimental group and the control group was statistically significant. Using Figure 1, we can detect the presence of the heterogeneity in our results. Here, the study’s confidence intervals are not overlapping and the SMD is not in close alignment, indicating heterogeneity in findings. The heterogeneity statistic, i.e., Q (Cochran’s Q, testing for heterogeneity) confirms the presence of heterogeneity in the SMD (Q_(__11)_=187.98, p<0.01). A nonsignificant p-value indicates little heterogeneity based on statistical analysis. A simple approach is to observe the relationship between the Q statistic, which is based on the χ^2^test, and its degrees of freedom (df).  If the statistic Q is greater than its df, one can conclude that there is statistical heterogeneity in the results. Here, the statistic Q is greater than its df, indicating the presence of heterogeneity. However, the Q statistic may provide a misleading measure of heterogeneity, because when the number of studies is small, the presence of statistical heterogeneity may be detected. When the number of studies is large, the presence of statistical homogeneity is not detected. Because of this, an effective approach to analysis, which is independent of the number of studies in the meta-analysis, is to observe the value of Higgins’s statistic I^2^to provide a richer and better picture of the presence of heterogeneity. Higgins’s statistic I^2^indicates the percentage of heterogeneity that is related to differences between studies (overall variations across studies), not random error.  It ranges from 0 % to 100%.  A high percentage of I^2^indicates a high degree of heterogeneity and a low percentage of I^2^indicates a low degree of heterogeneity.^7^ I^2^values of 25%, 50%, and 75% are considered as low, moderate and high heterogeneity, respectively. In this meta-analysis, the percentage of I^2^is large, i.e., 94.14%, indicating the presence of heterogeneity in the meta-analysis of the results.   Scholars using meta-analysts use two statistical models in the analysis, i.e., a fixed effects model and a random effects model. However, the heterogeneity of effects across studies can confuse the analyst as to which of these models should be used in the meta-analysis of studies. Below, these two models are discussed.

### Fixed-effect and random-effects models

Two statistical models are used for meta-analysis, the fixed-effect, and the random-effects models. The assumption associated with the fixed-effect model is that all studies in the meta-analysis have a common effect size (a low heterogeneity). However, in many systematic reviews, it is very unlikely for all studies to share a common true effect size in the real world because the true mean differs from one study to the next. If this is the case, we do not use the fixed-effect model to do a meta-analysis of a set of studies. Instead, we apply the random- effects model in order to estimate the mean of a distribution of effect sizes across all studies.^4^ Put simply, when statistical heterogeneity is low, a fixed-effect model is likely to be appropriate. When statistical heterogeneity is high, i.e., the results are more varied, a random effects model is likely to be appropriate. A further approach, which is called sensitivity analysis, is to run both statistical models and then detect how ‘sensitive the results of an analysis are to changes in the way the analysis was done’.^9^ If the effect sizes differ, the random-effects model would be favoured. Figure 2 shows the results based on a fixed-random effect using the same data based on the random-effects model (see Figure 1). Now we are in a position to compare Figure 1 with Figure 2. First, in the random-effects model, the confidence interval for the summary effect is wider than for a fixed-effects model, which is reflected in the diamond shape, resulting in a less precise total effect size.^10^ Second, in the random-effects model, the study weights are more or less the same. Studies with large sample sizes lose their influence in this model, while studies with low sample sizes gain influence.^4^

### Subgroup Analysis

It is necessary to identify the factors that yield effect size heterogeneity. For instance, in experimental studies, effect size heterogeneity could be studied for a specific independent variable, e.g., gender, the duration of an intervention or methods of assignment (random and non-random assignment). Subgroup analysis (sometimes called moderator analysis) is conducted to identify the potential source of effect size heterogeneity.  For example, consider the effect of simulation-based training on student performance. Here, we could run a heterogeneity test based on the sampling strategy (random vs. non-random) to explore moderating effects on effect sizes.  Sometimes effect size heterogeneity is rooted in lower-quality studies. Therefore, a sensitivity analysis of rigorous studies and non-rigorous studies shows whether or not the results are changed in relation to the rigor of the studies used in the meta-analysis. Sometimes effect size heterogeneity is rooted in different study designs, e.g., experimental studies and quasi-experimental studies. Conducting sensitivity analyses would help to identify the determinants of effect size heterogeneity.^9^ It has been argued the best approach to identify whether the heterogeneity effect size for different subgroups is statistically significant from the overall effect size using a heterogeneity test.  Therefore, in the forest plot, the no effect line is set at the overall effect size, not zero, to detect whether a subgroup confidence interval touches the no effect line from the total effect size.^11^

## Conclusions

This guide was intended to provide the reader with a brief introduction as to how to rigorously conduct a meta-analysis, especially for those who are new to the methods of meta-analysis. Overall, the purpose of the meta-analysis (which may complement a systematic review) is to systematically aggregate and statistically analyse the results of multiple studies to reach the total overall effect size (if the outcome is continuous), odds ratio or relative risk (if the outcome is dichotomous). Using a meta-analysis, analysts may obtain a clear picture of the effectiveness of an intervention because the statistical power of the study is increased by combining effect sizes across all studies. A forest plot provides useful information to assess the effects of variables of interest. To assess the amount of variation between the sample estimates, the statistical homogeneity tests are conducted, e.g., Cochran’s Q test and Higgins’s I^2^. These tests reveal the existence of heterogeneity between the sample estimates, but Higgins’s I^2^ provides an accurate result of the heterogeneity. If heterogeneity existed, the random-effects model will be applied. If homogeneity does not exist, the fixed-effects model will be applied. If we fit the random-effects model, the confidence interval for the overall effect would be wider than the fixed-effect model. Subgroup analyses indicate how the intervention effect is studied between the subgroups of interest, e.g., different study designs (experimental studies vs. quasi-experimental designs).

Finally, systematic reviews and meta-analyses provide an objective approach of combining a body of results which are important resources for evidence-based medical education. Given this value, medical educators who are concerned with curriculum planning and revision should have a greater understanding of the foundations of systematic reviews and meta-analyses to critically aggregate, integrate, synthesise and condense a collective body of articles in medical education. By doing this, we will provide rigorous and objective evidence for monitoring and improving the quality of teaching, learning and assessment in medical education.

### Conflict of Interest

The author declares that they have no conflict of interest.
